# Safety evaluation of percutaneous intrauterine hydrodissection in microwave ablation of uterine fibroids adjacent to the endometrium

**DOI:** 10.3389/fonc.2026.1674171

**Published:** 2026-01-30

**Authors:** Zhenlong Zhao, Ying Wei, Jie Wu, Shiliang Cao, Na Yu, Yan Li, Wenjia Cai, Ming’an Yu

**Affiliations:** Department of Interventional Medicine, China-Japan Friendship Hospital, Beijing, China

**Keywords:** endometrium, hydrodissection, intrauterine, microwave ablation, uterine fibroid

## Abstract

**Objective:**

To assess the safety and efficacy of percutaneous intrauterine hydrodissection as a protective technique during microwave ablation (MWA) of uterine fibroids adjacent to the endometrium.

**Materials and methods:**

A retrospective analysis was conducted on patients who underwent MWA with the percutaneous intrauterine hydrodissection strategy. The distribution of isolating fluid, the protective effect of intrauterine hydrodissection, its influence on ablation efficacy, and potential complications were assessed.

**Results:**

A total of 32 female patients were enrolled in the present study. The median age of the patients was 45 years old, with the median target uterine fibroid maximum diameter of 6.9 cm. Thirteen patients presented with fibroid-related symptoms. No evidence of fluid reflux into the pelvic cavity was detected during and after ablation on either CEUS or CT imaging. No case of residual tumor, infection, endometriosis, intestinal injury or bladder injury was observed during follow-up. Symptom improvement was observed in 69.2% (9/13) patients. No patient reported infertility due to intrauterine adhesion or endometrial injury after ablation.

**Conclusion:**

Percutaneous intrauterine hydrodissection is a safe and effective method for protecting endometrium during MWA of uterine fibroids adjacent to the endometrium.

## 1 Introduction

Uterine fibroids are a common benign clinical finding among women in reproductive age, with a prevalence of 70%-80% (1). Most fibroids are asymptomatic. However, larger fibroids—particularly those compressing the endometrium or submucosal fibroids—may cause abdominal distension, menorrhagia, infertility, or even anemia (2). Traditional treatments include open myomectomy, laparoscopic myomectomy, or hysteroscopic myomectomy. However, these procedures require myometrial incisions, potentially leading to intra-abdominal adhesions, uterine scarring, and an increased risk of uterine rupture during pregnancy (3). Additionally, fibroid recurrence may necessitate reoperation, which carries higher technical difficulty and complication rates. Therefore, less invasive alternatives are needed.

In recent years, thermal ablation techniques, such as high-intensity focused ultrasound (HIFU), radiofrequency ablation (RFA) and microwave ablation (MWA), have emerged as clinical options for fibroid treatment. Although HIFU is noninvasive, it demonstrates lower therapeutic efficacy compared to MWA and RFA (4). A significant concern with MWA of endometrium-compressing fibroids is that unprotected ablation may damage the endometrium, potentially resulting in complications such as scar pregnancy, menstrual disorders, or amenorrhea. Recent study has shown that intrauterine instillation of chilled saline can protect the endometrium during MWA of International Federation of Gynecology and Obstetrics (FIGO) type 1–3 uterine fibroids (5). Generally, there are two methods to protect the endometrium during MWA for uterine fibroids – placing a catheter in the uterine cavity and intraoperatively perfusing the uterine cavity with normal saline, or placing a core needle percutaneously into the uterine cavity and injecting normal saline. However, this procedure, similar to hysterosalpingography, carries potential risks of fluid reflux into the pelvic cavity, which may lead to endometriosis or infection (6, 7). Up to now, there is no research on the distribution of isolating fluid during percutaneous intrauterine hydrodissection to clarify this concern.

In present study, continuous intrauterine hydrodissection using normal saline with contrast agent to monitor the distribution of the isolating fluid was employed. All the enrolled patients underwent percutaneous intrauterine hydrodissection. The ablation efficacy for endometrium-compressing fibroids, the distribution pattern of the isolating fluid, and its effectiveness in endometrial protection was evaluated. This approach aims to provide a practical and safe endometrial protection strategy for MWA of such fibroids.

## 2 Materials and methods

Written informed consent was obtained from each patient before the ablation procedure. The patients consented to publishing their examination results and radiological images anonymously, and written informed consent for publication of their data was waived by the ethics committee of China-Japan Friendship Hospital.

### 2.1 Patients

From September 2020 to February 2025, patients underwent MWA for uterine fibroids at our center were retrospectively analyzed. The inclusion criteria were as follows: (1) non-menopausal women of reproductive age; (2) uterine fibroid confirmed by biopsy or having typical signs on two imaging examinations; (3) a maximum diameter exceeding 5 cm or symptomatic presentation such as abdominal distension and heavy menstrual bleeding (HMB); (4) percutaneous intrauterine hydrodissection strategy was used during the ablation procedure. The exclusion criteria were: (1) the distance between the fibroid and endometrium over 5 mm or not compressing the endometrium; (2) history of allergic reactions to iodine-based or ultrasound contrast agents; (3) patients who had surgery history; (4) incomplete data.

Following the NICE definition, HMB is defined as “excessive menstrual blood loss which interferes with a woman’s physical, social, emotional and/or material quality of life” (8).

### 2.2 Preablation assessment

Ultrasound imaging was performed using a 2.5–8 MHz convex array probe for both guiding the puncture and the imaging assessment. The three-dimensional diameters of the target fibroid, uterine size, and endometrial thickness were measured and recorded.

### 2.3 Intrauterine hydrodissection procedure

Prior to hydrodissection, local anesthesia was administered via subcutaneous injection of 1% lidocaine at the puncture site, as well as the peritoneum anterior to the uterus. An 18-gauge core needle connected to an extension tube and syringe was inserted with the needle tip positioned at the center of uterus cavity. When the uterine cavity was difficult to visualize clearly, normal saline was injected at the approximate location, and the position of the needle tip was adjusted based on diffusion pattern of the normal saline. When the needle tip was within the myometrium, the myometrium swelled, and no anechoic area were forming; when the needle tip was within the mucosa, the mucosa swelled, and the liquid could sometimes diffuse into the adjacent uterus cavity; and only when the needle tip was inside the uterine cavity, the anechoic isolating band within the cavity could be clearly visualized. For fibroids adjacent to the intestine, an additional core needle for artificial ascites was placed.

### 2.4 MWA procedure

The ablation procedure was performed by three radiologists, each with a minimum of 5 years of experience in MWA of uterine fibroids. The patient underwent bowel preparation before ablation and maintained at least six-hour fast. Following abdomen sterilization and intrauterine hydrodissection (as per the above protocols), a 0.5% lidocaine mixture was injected at the ablation antenna puncture point as well as the peritoneum anterior to the uterus for local anesthesia.

MWA was conducted under ultrasound guidance using one or two cooled MWA antennas with a 1.1-cm active tip (Intelligent Basic Type Microwave Tumor Ablation System, Nanjing ECO Microwave System, Nanjing, China). The antenna tip was placed 2–5 mm away from the corresponding fibroid capsule. The power was set at 40–50 W. Normal saline was continuously injected during ablation to maintain the intrauterine isolating distance of at least 5 mm. If the fibroids are pressing against the endometrium and the distance could not be achieved, the continuous flow of isolating fluid was maintained. After ablation, 30 ml normal saline supplemented with about 50 μL of Sonazoid contrast agent was injected through hydrodissection needle, and intrauterine contrast-enhanced ultrasound (CEUS) was performed to observe whether contrast agent was present in the pelvic cavity. If the imaging was suboptimal, an additional 30 mL of saline containing 5 mL of iodixanol was injected into the uterine cavity, followed by immediate CT scanning to assess whether contrast agent had reflux into the pelvic cavity. Two minutes after ablation, intravenous CEUS was performed to evaluate the ablation efficacy. Complete ablation was defined as the non-enhancement ablation zone entirely cover the target fibroid. Complications were observed and recorded during and after the procedure according to prior reports on ablation for uterus fibroids (9). The complications related to uterine fibroid ablation included hemorrhage, infection, intestinal tract or bladder injury, permanent menstrual disorder and post-ablation infertility.

### 2.5 Postablation assessment and follow-up visit

Technical success (complete ablation of uterine fibroid) was defined as the complete absence of enhancement on intravenous CEUS immediately after each procedure. All patients underwent follow-up examinations every 3 months during the first year, and every 6 months thereafter. Conventional ultrasound was used for follow up evaluation, including measurement of the ablation zone dimensions and assessment of blood flow using color Doppler flow imaging. Symptom improvement was defined as improvement of the presenting symptoms (either HMB or abdominal distension) during follow-up visits. Residual tumor was defined as the ablation zone failed to completely cover the original fibroid during follow up. At each follow-up visit, patients were asked whether their menstrual cycles were regular and whether any necrotic tissue had been expelled from the vagina.

### 2.6 Statistical methods

Statistical analyses were performed using SPSS software, version 24.0 (IBM, Armonk, NY, USA). Data are presented as mean ± standard deviation (SD) for normally distributed variables, while median and interquartile range (IQR, 25th-75th percentiles) were used for non-normally distributed data.

## 3 Results

### 3.1 Demographic and nodule characteristics

**Figure 1:**
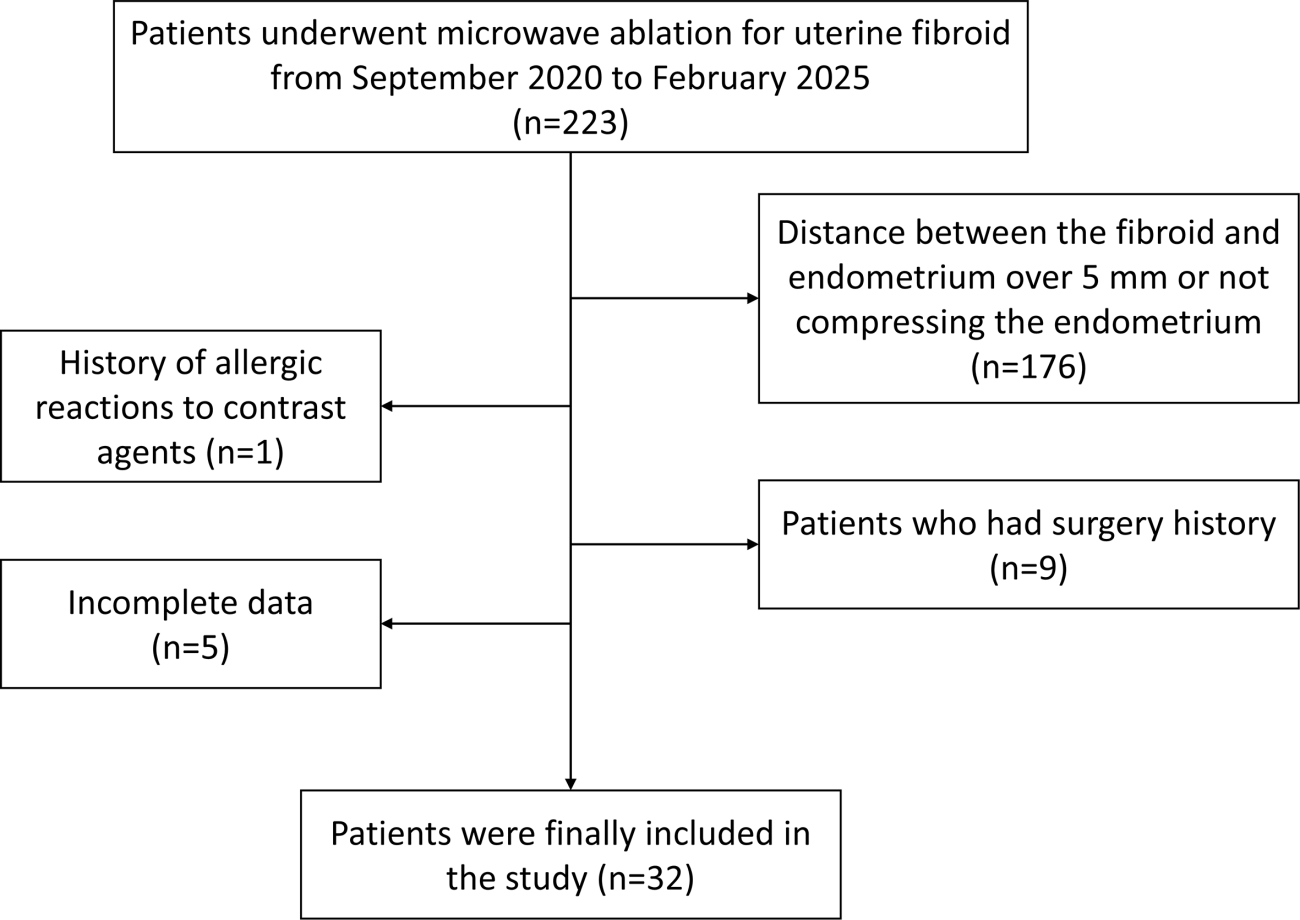
Study flow chart.

A total of 32 female patients were enrolled in the present study. The flow chart of patient selection is shown in **Figure 1**. The median age of the patients was 45 (25-75% IQR 39-47.25, age range 29-51) years old. The median maximum diameter of the target uterine fibroids was 6.9 (25-75% IQR 5.7-7.8, age range 3.7-12.6) cm. The fibroid type according to FIGO classification included type-1 in 3 cases and type 2–5 in 29 cases (10). Of the 32 patients, 40.6% (13/32) presented with symptoms due to uterine fibroids. Menorrhagia was reported in 31.3% (10/32) patients, and abdominal distension in 15.6% (5/32) patients.

### 3.2 Hydrodissection result and adverse effects

**Figure 2:**
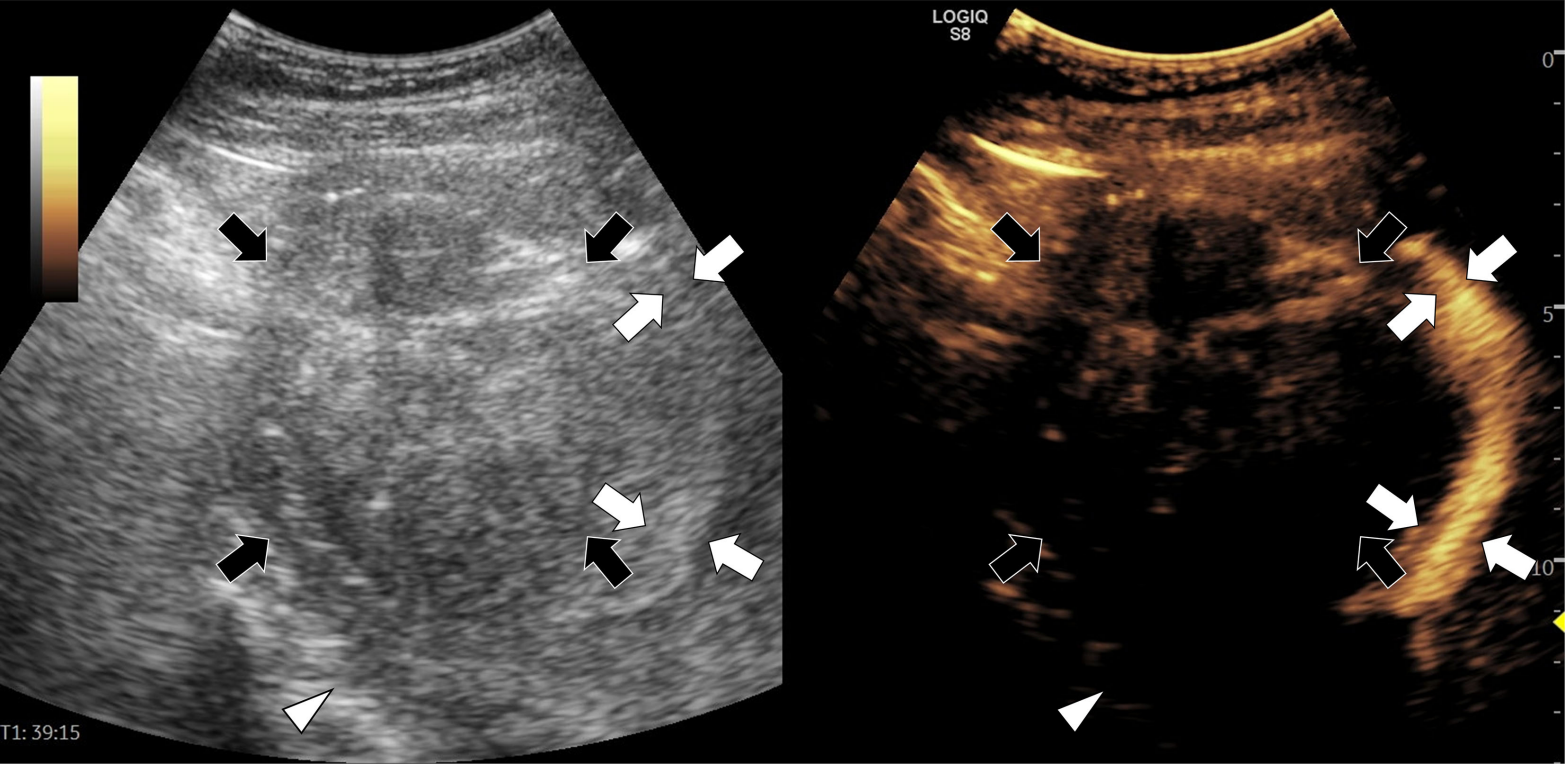
A 50-year-old woman with a uterus fibroid compressing the endometrium underwent microwave ablation. Intrauterine hydrodissection was performed before ablation. The uterus cavity (white arrows) is filled with normal saline containing contrast agent, and the endometrium is compressed by the fibroid (black arrows). No contrast agent was observed in the pelvic cavity (white triangle).

**Figure 3:**
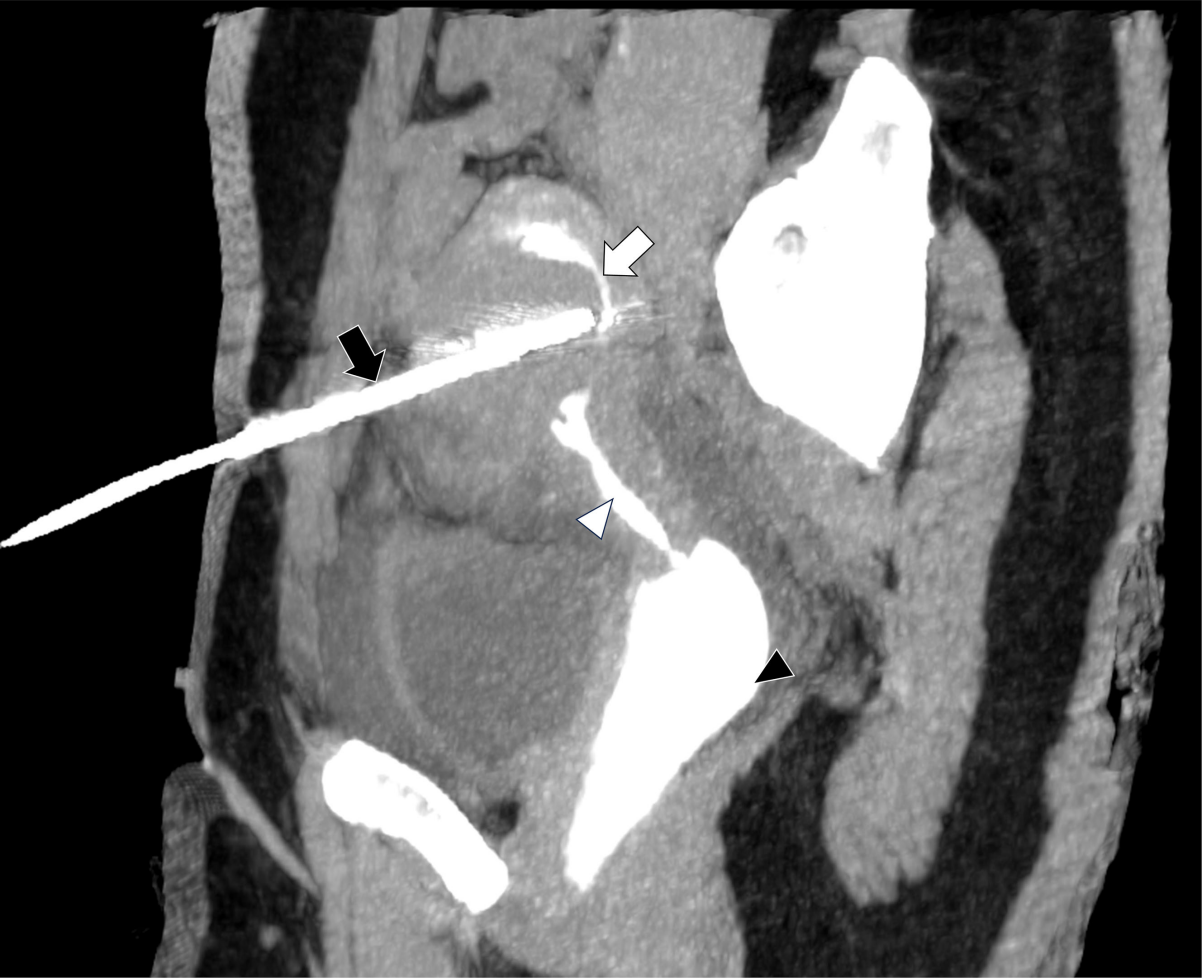
The sagittal and maximum intensity projection contrast-enhanced CT image of uterine cavity after fibroid ablation for a 50-year-old woman. The uterus cavity (white arrow), cervix (white triangle) and vagina (black triangle) is filled with normal saline containing contrast agent. The ablation antenna (black arrow) did not reach the uterus cavity. No contrast agent was observed in the pelvic cavity.

Intrauterine hydrodissection was successfully performed in all enrolled patients. The hydrodissection procedure time was 0.5–2 minutes. The patients only reported various degrees of tension pain during isolating fluid injection. Four patients (4/32, 12.5%) had hemorrhages in isolating band, and stopped spontaneously without intervention. During the procedure, the isolating fluid accumulated in the vaginal fornix. After needle removal following the procedure, patients showed no signs of uterine artery bleeding or hematocoelia. The isolating fluid flowed out of the vagina when patients assumed a sitting position after the procedure. No evidence of fluid reflux into the pelvic cavity was detected on either CEUS or CT imaging (**Figures 2** and **3**). Ultrasound examination within 24 hours after the procedure showed no fluid collection or increase in pre-existing small amounts of fluid in the pelvic cavity. The patients showed no signs of suspicious pelvic inflammation or symptoms and manifestations of endometriosis.

### 3.3 Ablation outcome

The complete absence of enhancement on intravenous CEUS was observed in all target fibroids after ablation. The technical success rate was 100%. Normal endometrium enhancement was confirmed by CEUS in all cases after ablation. During the follow-up period, no case with residual tumor, infection or endometriosis was encountered. There was no case of intestinal tract or bladder injury, either. All patients maintained stable vital signs after the procedure. Follow-up assessments showed that the uterine fibroid-related symptoms resolved in 69.2% (9/13) patients. No patient reported infertility due to intrauterine adhesion or endometrial injury after ablation.

## 4 Discussion

Minimally invasive methods, including HIFU, RFA and MWA, have been employed for several years in the treatment of uterine fibroids, offering similar treatment effectiveness and fewer complications compared with surgery (9, 11, 12). However, many uterine fibroids often develop insidiously, and by the time the patients experience symptoms such as abdominal distension and menstrual irregularities, the fibroids are usually already large in size and compress the endometrium. During ablation for such fibroids, there is an increased risk of endometrium injury, which can lead to complications such as uterine perforation, scar pregnancy, and infertility. In a recent study, 42.8% patients had endometrial impairment after HIFU procedure (13). Therefore, endometrium protection is essential during thermal ablation of uterine fibroids adjacent to the endometrium.

One of the methods for intrauterine hydrodissection method involves the retrograde insertion of a pediatric catheter through the vagina and cervix. The catheter is fixed at the internal cervical os using a lateral balloon, followed by isolating fluid injection during ablation. Although this approach is non-invasive, retrograde manipulation carries the risk of infection and requires preoperative assessment of vaginal cleanliness. Additionally, balloon occlusion of the cervical os during fluid injection may lead to fluid reflux into the pelvic cavity.

Through extensive clinical practice, our team has found that percutaneous insertion of a core needle directly into the uterine cavity is a simple and effective procedure. Compared to traditional methods, this method avoids retrograde vaginal manipulation. However, potential risks such as retrograde fluid reflux into the pelvis, infection, endometrial implantation, or procedural complications require further investigation.

The results of the present study demonstrate that percutaneous intrauterine hydrodissection is a simple and practical method, requiring only 0.5–2 minutes of procedure time, with a 100% success rate. Minor intrauterine bleeding occurred in 12.5% of patients, but no hematocoelia was observed. This outcome may be attributed to precise ultrasound guidance and the relatively broad area of the uterine cavity, which facilitates easier needle insertion. Additionally, color Doppler flow imaging enabled clear visualization of major vessels along the needle path, minimizing the risk of vascular injury. Postoperative CEUS or CT confirmed no fluid reflux into the pelvic cavity, indicating that all fluid was expelled through the cervix and vagina. No new pelvic fluid accumulation or increase in pre-existing fluid was observed, and there were no cases of pelvic infection or endometriosis. These results indicate that percutaneous hydrodissection with mild-pressure fluid injection ensures complete isolating fluid expulsion through the vagina without reflux. The pressure in interstitial and isthmic portions of the fallopian tubes might be higher than that in cervix and vagina, which make the isolating fluid flow from the uterine cavity toward the cervix, and have the dominant role in directing fluid flow. Additionally, the pressure in the pelvic cavity is greater than atmospheric pressure, and this pressure difference also favors fluid expulsion through the vagina rather than reflux into the pelvic cavity. In contrast, transvaginal insertion of a urinary catheter may increase uterine cavity pressure due to the blocking effect of the balloon, causing fluid reflux into the pelvic cavity. During hysteroscopy, fluid is injected under even higher pressure to optimally distend the uterine cavity for surgical visualization and manipulation. The infusion rate during intrauterine hydrodissection was approximately 30–60 ml/min, significantly slower than the 200 ml/min used during hysteroscopy (14). This increased pressure consequently elevates the probability of pelvic fluid reflux.

Follow-up results showed no occurrence of endometriosis or abdominal wall endometrial implantation. These results indicate that although percutaneous intrauterine hydrodissection involved needle puncture into the uterine cavity with possible slight damage to the endometrium, this damage was minor compared with surgery, and could not cause needle tract implantation when withdrawing the needle.

Typically, uterine fibroids have pseudocapsule (15), which could confine the heat within the fibroid during ablation while minimizing the cooling effect from intrauterine hydrodissection. In present study, there was no case of residual tumor, indicating that ablation efficacy was not influenced by intrauterine hydrodissection. The uterine fibroid-related symptoms resolved in 69.2% patients, and no patients reported infertility due to intrauterine adhesion or endometrial injury after ablation. These results demonstrate that percutaneous intrauterine hydrodissection could protect the structure and function of the endometrium while achieving complete ablation of the fibroids, and is an effective technique worthy of promotion.

While this is the first study to document isolating fluid distribution during percutaneous intrauterine hydrodissection using CEUS and CT, 100% technical success rate, and confirmed absence of pelvic fluid reflux, the present study has several limitations. First, the absence of a control group without intrauterine hydrodissection. Second, this is a retrospective study with a small sample size, single-center design, and limited statistical power, and potential selection bias exists. Third, lack of further hysteroscopic verification of intrauterine adhesions. Fourth, endometrial function was assessed only through menstruation outcomes, which might underestimate minor endometrial impairments.

## 5 Conclusions

Percutaneous intrauterine hydrodissection is a safe and effective method for protecting endometrium during MWA of uterine fibroids adjacent to the endometrium. This technique expands the applicability of MWA to endometrium-compressing fibroids while preserving endometrial function and preventing fluid reflux-related complications such as infection and endometrial implantation.

## Data Availability

The datasets presented in this article are not readily available due to patient privacy and confidentiality constraints. Requests for data access should be directed to the corresponding author and are subject to review by the institutional ethics committee.
